# Bosentan as adjunctive therapy in neonates with congenital diaphragmatic hernia-associated pulmonary hypertension: a case series

**DOI:** 10.1007/s00431-025-06019-6

**Published:** 2025-02-13

**Authors:** Aster De Vadder, Lotte Lemloh, Bartolomeo Bo, Lennart Hale, Neil Patel, Andreas Mueller, Florian Kipfmueller

**Affiliations:** 1 Department of Neonatology and Pediatric Intensive Care, Children’s Hospital, University of Bonn, Venusberg-Campus 1, 53127 Bonn, Germany; 2 Department of Neonatology, The Royal Hospital for Children, Glasgow, UK

**Keywords:** Bosentan, Newborn, Congenital diaphragmatic hernia, Pulmonary hypertension, Endothelin-1 receptor antagonist

## Abstract

Congenital diaphragmatic hernia (CDH)-associated pulmonary hypertension (PH) is associated with high morbidity and mortality. Pulmonary vasodilative management is challenging and some patients with CDH are unresponsive to inhaled nitric oxide or sildenafil. Bosentan, an enterally-administered endothelin-1 receptor antagonist, reducing pulmonary vascular resistance may play a role in the treatment of CDH-PH. The aim is to evaluate the efficacy and safety of bosentan as an adjunctive therapy for CDH-PH. We report a case series of all CDH neonates who received oral bosentan as an adjunctive therapy for treatment of PH between 2013 and 2021 at our institution. Bosentan was administered at a median enteral dose of 2 mg/kg/day. Main outcomes were improved PH severity on echocardiography, oxygenation, and respiratory support after starting bosentan. Patients were compared according to improvement in PH after 1 week of treatment (responder vs. non-responder). Fifty CDH neonates received oral adjunctive bosentan therapy. Survival to discharge was 58%. Improved PH was observed in 54 and 72% of patients after 1 and 2 weeks respectively (*p* < 0.001). Respiratory status ameliorated significantly after 2 weeks compared to baseline, with a reduction of ECMO treatment from 30 to 0% and an increase in patients receiving non-invasive or no respiratory support from 18 to 40%. Oxygenation did not improve over 2 weeks, possibly biased by the changes in the respiratory status and other contributing factors to the pathophysiology of CDH.

*Conclusion*: Bosentan is effective in the treatment of neonates with CDH-PH and was associated with improved PH severity and respiratory status. Adverse effects were minimal and consistent with previous studies.

**Table:** 

**What Is Known:** *• CDH neonates frequently suffer from pulmonary hypertension with inconclusive evidence regarding the benefit of pulmonary vasodilator treatment.* *• Increased endothelin-1 plasma levels have been associated with poor outcome in CDH neonates, however, there is minimal data on the use of endothelin receptor blockers, such as bosnetan, in this population.* **What Is New:** *• This case series of 50 CDH neonates receiving bosentan demonstrates an improvement in PH severity based on echocardiographic assessment in 54% within one week of treatment.* *• Respiratory support modus (i.e. ECMO, mechanical ventilation, CPAP) improved significantly within two weeks of bosentan treatment in responders and non-responders.*

**What Is Known:**

*• CDH neonates frequently suffer from pulmonary hypertension with inconclusive evidence regarding the benefit of pulmonary vasodilator treatment.*

*• Increased endothelin-1 plasma levels have been associated with poor outcome in CDH neonates, however, there is minimal data on the use of endothelin receptor blockers, such as bosnetan, in this population.*

**What Is New:**

*• This case series of 50 CDH neonates receiving bosentan demonstrates an improvement in PH severity based on echocardiographic assessment in 54% within one week of treatment.*

*• Respiratory support modus (i.e. ECMO, mechanical ventilation, CPAP) improved significantly within two weeks of bosentan treatment in responders and non-responders.*

## Introduction

Congenital diaphragmatic hernia (CDH) is a neonatal condition caused by a diaphragmatic defect allowing abdominal organs to herniate into the thorax, leading to aberrant pulmonary and cardiovascular development. It affects approximately 1:2500 to 1:3000 live births [1]. Despite advances in treatment over the past decade, mortality remains high ranging from 30 to 50% [2]. This is mainly because of the resulting hypoplasia of both lungs, abnormal development of the pulmonary vasculature with increased thickening of the pulmonary vascular smooth muscle cells, hypoplasia of the pulmonary vasculature, abnormal vascular responsiveness and cardiac dysfuntion [3–5], altogether resulting in pulmonary hypertension (PH) with elevated right-heart pressure, circulatory shunting, poor ventilation and decreased oxygenation [6]. Right and left ventricle (RV and LV, respectively) dysfunction are also significant factors contributing to the pathophysiology of CDH-PH and determining the correct treatment. LV dysfunction may lead to pulmonary venous hypertension, causing pulmonary capillary syndrome (post-capillary PH) with alveolar oedema impacting lung compliance and gas transfer. This often appears to be an acute postnatal phenomenon, most severe immediately after birth and improving in the first week after life, whereas RV dysfunction frequently leads to impaired pulmonary blood flow and oxygenation (pre-capillary PH) and may persist until the post-operative period. Pre-capillary PH should primarily be treated with pulmonary vasodilators to decrease pulmonary vascular resistance (PVR), whereas pulmonary vasodilatation in post-capillary PH may be detrimental, since its increased pulmonary return may overload the LV, worsening LV dysfunction. On the other hand, suboptimal lung recruitment and alveolar overdistention, causing increased PVR and RV afterload, may increase and exacerbate RV dysfunction, therefore reducing LV preload and worsening LV function and systemic output [7]. The medical management of CDH-PH therefore remains challenging and is often not supported by evidence from multicentre randomized controlled trials (RCTs) [1]. In the past, inhaled nitric oxide (iNO) and intravenous sildenafil have been widely used to treat CDH-PH, but not all patients respond sufficiently. Although, mechanisms leading to an insufficient response to pulmonary vasodilators are poorly investigated, the underlying pathophysiology of the lung, insufficient lung recruitment, hyperinflation and differences in cardiac dysfunction are paramount. Therefore, newer alternatives or adjunctive therapies are being explored [8, 9]. The endothelin pathway may play an important role, making it a focus of current research. Endothelin-1 (ET-1), a potent pulmonary vasoconstrictor, binds primarily to endothelin-A (ET-A) and -B (ET-B) receptors. ET-A receptor stimulation promotes vasoconstriction, while ET-B receptors mediate vasodilation [6, 10]. Studies have shown significant overexpression of ET-A and ET-B receptors and increased levels of circulating ET-1 in patients with PH [5, 11–13]. Additionally, circulating ET-1 levels correlate positively with disease severity and decrease as the disease resolves [12, 14]. These findings suggest that dysregulation of ET-A receptors and elevated ET-1 levels may contribute to CDH-PH. Bosentan, an antagonist of both ET-A and ET-B receptors with higher affinity for ET-A receptors, has been shown to have a beneficial effect and favourable safety profile in adults and pediatric patients with pulmonary arterial hypertension [15, 16]. Bosentan has also been proposed by Fortas et al. as a third line therapy in a rational clinical algorithm for refractory PH [17]. However, research on bosentan’s effect in neonates is limited with PH causes varying widely in these studies [10, 15, 16, 18–22]. No studies have specifically investigated the effect of bosentan in infants with CDH-PH. Here, we present a case series of our clinical experience with oral adjunctive bosentan treatment in infants with CDH-PH.

## Materials and methods

### Study design and ethical approval

**Fig. 1:**
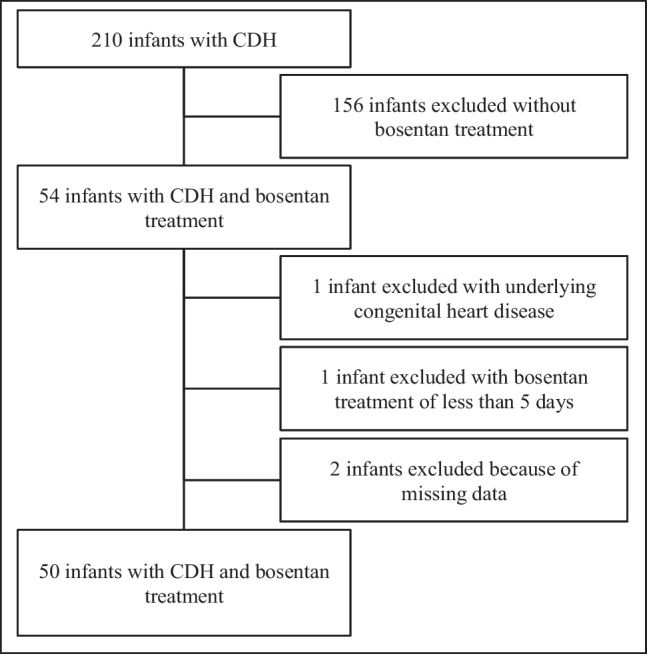
Flow diagram of congenital diaphragmatic hernia (CDH) cohort

We retrospectively reviewed a case series of infants with CDH receiving bosentan treatment at the University Children’s Hospital of Bonn between February 2013 and March 2021. Infants with an underlying congenital heart defect or with less than 5 days of bosentan treatment were excluded. Two infants were not included because of missing data (Fig. 1). The study was approved by the ethics committee of the Medical Center of the University of Bonn (No.: 200/22) and informed consent was waived due to the retrospective design of the study.

### Standard treatment protocol

At birth, all newborns were immediately intubated, and mechanical pressure-controlled ventilation was started, allowing permissive hypercapnia (PaCO_2_ 45–65 mmHg) with a pH of > 7.25. A low-pressure ventilation strategy was achieved with a positive end expiratory pressure (PEEP) of 2–4 mmHg, a positive inspiratory pressure (PIP) of < 26 mmHg, a breathing rate of 50–70 per minute and an inspiration time of 0.4 s. Modulation of ventilation was performed individually without formal protocol. High frequency oscillation ventilation (HFOV) was used as a late rescue ventilation. Initial inspired oxygen fraction (FiO_2_) was 1.0 and was adjusted to achieve a preductal oxygen saturation (SpO_2_) of > 90%. Inhaled NO of 20 parts per million (ppm) was started simultaneously. Use of extracorporeal membrane oxygenation (ECMO) followed criteria described by the CDH Euro Consortium Consensus [23]. There was no need for repeat ECMO therapy in this cohort of patients. Newborns were started on oral adjunctive bosentan treatment if PH was graded more than 2/3 of the systemic pressure using echocardiography despite existing therapy with intravenous sildenafil or iNO, when there was a persistent elevated need of oxygen supplementation without possible other explanation on radiographic images, or when the oxygenation index was more than 15. Bosentan was started at a median dose of 1 mg/kg/d every 12 h. Sildenafil and iNO were continued after starting bosentan therapy. With improving PH, iNO was taken out firstly. Systemic pressers (norepinephrine and vasopressin as first and second line, respectively) were used to achieve a normal blood pressure. In context of LV dysfunction, we aimed to accept a lower blood pressure and wean patients of systemic pressers accordingly. Cardiac dysfunction was primarily treated with milrinone und dobutamine, in severe cases with levosimendan.

### Data collection and outcomes

Outcome data were collected for a period of 3 h before and 14 days after the start of enteral bosentan therapy. Baseline values were defined as data at the start of bosentan. The following monitoring and laboratory data were recorded: partial arterial pressure of oxygen (PaO_2_), fraction of inspired oxygen (FiO_2_), preductal oxygen saturation (SpO_2_), mean airway pressure (MAP), positive end-expiration pressure (PEEP), the oxygenation index (*OI*) calculated with the formula *OI* = (MAP × FiO_2_ × 100)/PaO_2_, the oxygenation saturation index (*OSI*) calculated with the formula *OSI* = (MAP × FiO_2_ × 100)/SpO_2_ and the saturation oxygen distending pressure index (*SOPI*) calculated with the formula *SOPI* = (PEEP × FiO_2_ × 100)/SpO_2_. Liver function tests (LFT) were recorded for the length of hospital stay after the start of bosentan. The primary outcome was defined as an improvement of at least one severity grade of PH on echocardiography 1 week after starting bosentan treatment. Neonates were divided into a responder (improvement of PH) and a non-responder group (no improvement or worsening of PH). Secondary outcome parameters were severity of PH after 2 weeks of treatment, at discharge and 12 months after discharge, the ratio of right ventricular diameter to left ventricular diameter (*RV*_*D*_/*LV*_*D*_) at 1 and 2 weeks after starting bosentan, and at discharge, duration of ECMO, mechanical ventilation, and respiratory support, improvement in oxygenation status (improvement of 20% in *OI/SOPI* after 1 week of treatment), and survival to discharge.

### Echocardiographic data and PH definition

Echocardiography was performed by a neonatologist specialized in neonatal echocardiography and retrospectively reviewed for the severity of PH at baseline, 1 and 2 weeks after the start of treatment with bosentan, at discharge and 12 months after discharge. The severity of PH was graded into three categories: mild PH (pulmonary arterial pressure (PAP) < 2/3 of the systemic systolic pressure), moderate PH (PAP of 2/3 to systemic systolic pressure) and severe PH (suprasystemic systolic pressure) [14]. This grading of PH was calculated including the ductus arteriosus flow pattern, the intraventricular septum position and the tricuspid valve regurgitation velocity. Additionally, the *RV*_*D*_/*LV*_*D*_ ratio was retrospectively measured as an indicator for PH severity (Cut-off *RV*_*D*_/*LV*_*D*_ ratio ≥ 1.0) [24]. As previously described, *RV*_*D*_ and *LV*_*D*_ were measured distal to the tricuspid and mitral annulus as a horizontal line from the endocardium of the *RV* and *LV* free wall to the endocardium of the interventricular septum perpendicular to the long axis. End diastole was defined as the frame with the maximum ventricular area corresponding to mitral valve closure and at the end of the *R* wave on electrocardiogram trace [25]. Qualitative assessment of *LV* function was obtained with each echocardiogram. Additionally, *LV* dysfunction was indicated by global or regional cardiac hypokinesia, fractional shortening ≤ 25%, ejection fraction ≤ 45% or *LV* output < 100 ml/kg/min. A global longitudinal strain (GLS) of the *LV* of ≥ − 16% was defined as demonstrating *LV* dysfunction. Persistent ductus arteriosus (PDA) shunt was defined as left-to-right, bidirectional, or right-to-left, but shunt direction was not used to guide bosentan treatment.

### Statistical analysis

SPSS Version 29 (IBM Corp. Armonk, NY) was used for statistical analysis. Continuous variables were described using median and interquartile range (*IQR*) and categorical variables were summarized as absolute number (*n*) and percentage. Comparison between groups was performed with Mann–Whitney-*U* test and Chi2 test for continuous and categorical covariates, respectively. A mixed model with patient-ID as random factor was used to assess significant changes in echocardiography data, respiratory status, oxygenation and ventilator settings. A *p*-value of < 0.05 was considered to indicate statistical significance between groups.

## Results

### Basic characteristics

Fifty-four of 210 CDH neonates (25.7%) were treated with bosentan between February 2013 and March 2021 at our institution. The final study sample consisted of 50 CDH neonates, after excluding four patients. Survival to discharge was 58% (29/50), and 37 infants (74%) were treated with ECMO. The group of responders consisted of 27 infants (54%) with an improved PH severity grade 1 week after starting bosentan, whereas 23 patients (46%) were classified as non-responder. Baseline characteristics are demonstrated in Table 1. Gender, gestational age, birth weight, proportion of prenatal diagnosis, side of the diaphragmatic hernia, defect size, rate of fetal endoscopic tracheal occlusion (FETO) and amount of an isolated diaphragmatic hernia were not significantly different between groups.

**Table 1:** Baseline characteristics at birth. Data is demonstrated as absolute numbers with percentages or median with interquartile range (*IQR*)

Variables	Responder *n* = 27	Non-responder *n* = 23	*P*-value
**Demographics**			
Gender (*n*)	15 (56%)	11 (48%)	0.589
Gestational age (weeks)	38.0 [36.6–38.6]	36.4 [34.9–38.1]	0.055
Birth weight (kg)	3.2 [2.7–3.3]	2.7 [2.1–2.9]	0.054
Prenatal diagnosis (*n*)	25 (93%)	21 (91%)	0.868
Left-sided CDH (*n*)	22 (81%)	19 (83%)	0.918
o/e LHR (%)	38.0 [30.5–43.3]	31.0 [28.5–39.0]	0.087
Intra-thoracic liver (*n*)	20 (74%)	21 (91%)	0.118
Isolated CDH (*n*)	18 (86%)	10 (57%)	0.686
FETO (*n*)	6 (22%)	6 (26%)	0.752

*CDH* congenital diaphragmatic hernia, *o/e LHR* observed-to-expected lung-to-head ratio, *FETO* fetal endoscopic tracheal occlusion

### Clinical course and treatment data

Data on concomitant therapies, baseline data and outcome are summarized in Table 2. The median age at starting bosentan was similar between groups (12.8 days vs. 11.5 days, *p* = 0.977), and the median dose of bosentan at baseline was in both groups 2.0 mg/kg/day. All neonates, except one where iNO was started later, were treated with iNO and sildenafil at the time of starting bosentan. Eighty-two percent and 66% of newborns received milrinone and dobutamine at baseline, whereas 56% and 42% were treated with norepinephrine and vasopressine, respectively, at the start of treatment. Diuretics and steroids were given to 92% and 32% of newborns, respectively, at the time of starting bosentan. We observed no significant difference in the use of the above-mentioned medications between responders and non-responders at baseline. Ninety percent (45/50) continued iNO after starting bosentan therapy and the median duration of iNO continuation was 20.5 days. All patients were continued on and discharged with sildenafil, except for 1 patient who was discharged with tadalafil. Twenty-four percent (7/29) of survivors were discharged with bosentan therapy. Seventy percent of the responders were treated with ECMO therapy, compared to 78% of the non-responders (*p* = 0.530). At baseline, we observed a similar number of patients in need of mechanical ventilation and on ECMO therapy in both groups. Bosentan was started while on ECMO therapy for 30.4% (7/23) of the non-responder and 29.6% (8/27) of the responder, whereas 43.5% (10/23) of non-responders and 40.7% of responders were started after ECMO therapy already stopped. For 1 patient in the non-responder group bosentan preceded ECMO therapy. GLS was similar between responders and non-responders at the start of bosentan treatment and after 7 days of treatment (Table 2). Overall, 39.1% of non-responders and 25.9% of responders had an abnormal GLS (≥ − 16.0%) at the initiation of bosentan (*p* = 0.230). A PDA was observed in 56.5% and 37.0% of non-responders and responders, respectively (*p* = 0.373; Table 2). Significantly fewer responders received bosentan at discharge when compared to non-responders (44% vs. 74%, *p* = 0.037). The duration of ECMO therapy, mechanical ventilation and hospital stay did not significantly differ between both groups. Elevation of liver function tests was seen in 8 neonates (16%) within an average of 32 days after starting bosentan treatment. The median length of hospital stay only calculated for surviving patients was 78 days, with a median of 66.4 days in the non-responder group and a median of 85.8 days in the responder group.

**Table 2:** Comparison between responder and non-responder in bosentan treatment, concomitant therapies, echocardiographic and outcome data. Data is demonstrated as absolute numbers with percentages or median with interquartile range (*IQR*)

Variables	Study population *n* = 50	Responder *n* = 27	Non-responder *n* = 23	*P*-value
**Concomittant therapies**				
Inhaled nitric oxide (*n*)	50 (100%)	27 (100%)	23 (100%)	1.000
IV sildenafil (*n*)	50 (100%)	27 (100%)	23 (100%)	1.000
ECMO (*n*)	37 (74%)	19 (70%)	18 (78%)	0.530
Operated (*n*)	48 (96%)	27 (100%)	21 (95%)	0.268
Patch repair (*n*)	45 (90%)	24 (89%)	21 (95%)	0.409
**Baseline data (treatment start)**				
*RV*_*D*_/*LV*_*D*_ ratio at baseline	1.2 [1.1–1.4]	1.2 [1.1–1.4]	1.1 [1.0–1.4]	0.300
ECMO at baseline (*n*)	15 (30%)	8 (30%)	7 (30%)	0.951
Mechanical ventilation at baseline (*n*)	40 (80%)	20 (77%)	20 (87%)	0.370
CPAP at baseline (*n*)	9 (18%)	6 (22%)	3 (13%)	0.405
Bosentan dose at baseline (mg/kg/d)	2.0 [1.9–2.2]	2.0 [1.8–2.2]	2.0 [1.9–2.4]	0.107
Age starting bosentan (days)	12.5 [7.4–19.2]	12.8 [6.0–19.5]	11.5 [8.0–19.3]	0.977
Inhaled nitric oxide (*n*)	49 (98%)	27 (100%)	22 (95.6%)	0.279
IV sildenafil (*n*)	50 (100%)	27 (100%)	23 (100%)	1.000
Milrinone (*n*)	41 (82%)	22 (81.5%)	19 (82.6%)	0.918
Dobutamine (*n*)	33 (66%)	17 (63%)	16 (69.6%)	0.627
Norepinephrine (*n*)	28 (56%)	16 (59.3%)	12 (52.3%)	0.618
Vasopressin (*n*)	21 (42%)	10 (37%)	11 (47.8%)	0.446
Diuretics (*n*)	46 (92%)	26 (96.3%)	20 (87%)	0.230
Steroids (*n*)	16 (32%)	7 (25.9%)	9 (39.1%)	0.323
Persistent ductus arteriosus (*n*)	23 (46%)	10 (37%)	13 (56.5%)	0.373
Global longitudinal strain (GLS) > − 16% (*n*)	16 (32%)	7 (25.9%)	9 (39.1%)	0.230
**Echocardiography**				
No persistent ductus arteriosus (PDA) (*n*)	27 (54%)	17 (63%)	10 (43.5%)	0.305
PDA: Left-to-right shunt (> 75%) (*n*)	16 (32%)	7 (25.9%)	9 (39.1%)	0.514
PDA: bidirectional shunt	7 (14%)	3 (11.1%)	4 (17.4%)	0.356
*RV*_*D*_/*LV*_*D*_ ratio after 1 week	1.0 [0.9–1.2]	1.0 [0.9–1.2]	1.0 [0.9–1.3]	0.156
*RV*_*D*_/*LV*_*D*_ ratio after 2 weeks	0.9 [0.9–1.1]	0.9 [0.8–1.1]	1.0 [0.9–1.3]	**0.028**
*RV*_*D*_/*LV*_*D*_ ratio at discharge	0.9 [0.8–1.1]	0.9 [0.8–1.0]	0.9 [0.9–1.3]	0.303
*LV-GLS* at baseline	− 17.6 [− 20.3–15.2]	− 17.7 [− 19.7–15.3]	− 17.6 [− 20.5–15.9]	0.819
*LV-GLS* after 1 week	− 18.8 [− 20.5–16.7]	− 18.9 (− 20.1–17.5)	− 18.1 (− 20.7–15.9)	0.582
**Outcome**				
Discharge with bosentan (*n*)	29 (58%)	12 (44%)	17 (74%)	**0.037**
Mortality (*n*)	21 (42%)	9 (33%)	12 (52%)	0.183
Mechanical ventilation (days)	27.9 [16.0–38.5]	26.8 [14.7–37.0]	27.9 [14.1–45.3]	0.771
Length of hospital stay (days)	72.4 [43.2–126.6]	80.0 [3.0–152.3]	65.5 [35.2–107.6]	0.209
ECMO duration (days)	8.0 [3.0–14.0]	6.0 [1.0–16.3]	8.0 [3.0–14.0]	0.848
LFT > 3 × ULN (*n*)	8 (16%)	3 (11%)	5 (22%)	0.312

*CPAP* continuous positive airway pressure, *IV* intravenous*, ECMO* extracorporeal membrane oxygenation. *RV*_*D*_*/LV*_*D*_* ratio* the ratio of right ventricular diameter to left ventricular diameter, *LFT* liver function test, *ULN* upper limit of normal, *PDA* persistent ductus arteriosus, *GLS* global longitudinal strain

P-values presented in bold indicate p<0.05

### Primary and secondary outcome

**Fig. 2:**
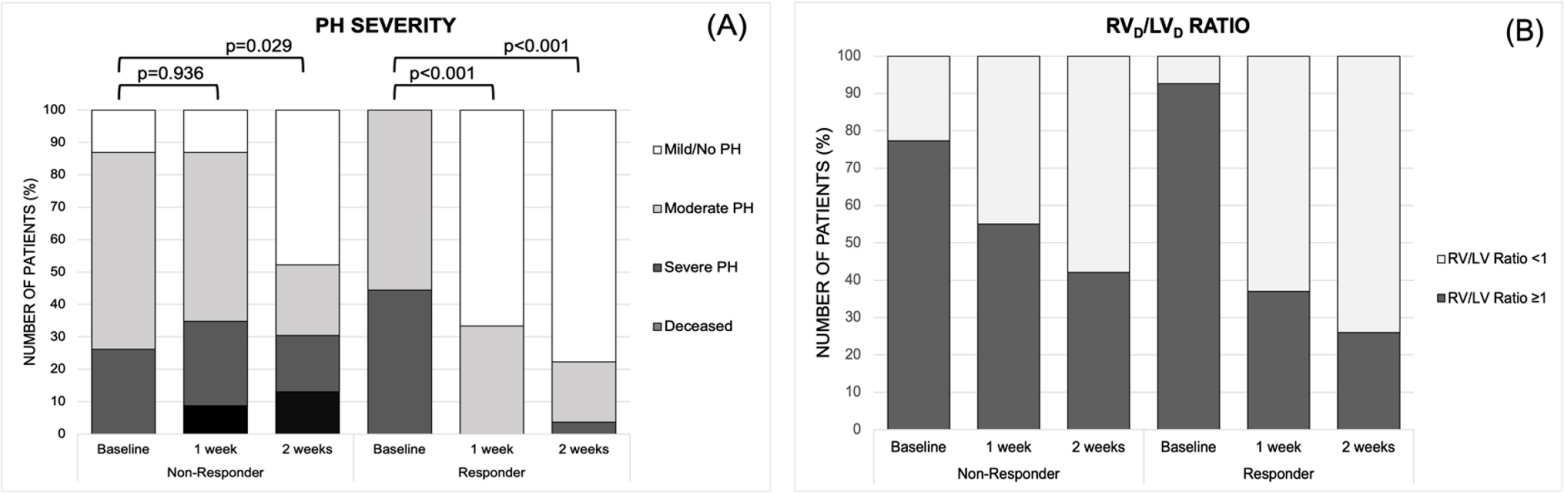
**A** Pulmonary hypertension severity during 2 weeks after commencing bosentan. PH, pulmonary hypertension. *P*-values indicate differences in PH severity compared to baseline. **B** Effects of bosentan on the *RV*_*D*_/*LV*_*D*_ ratio. *n* = 22 at baseline, *n* = 20 after 1 week and *n* = 19 after 2 weeks in the group of non-responders. *n* = 27 in the group of responders. Declining study population in the group of non-responders is due to missing echocardiographic data for 1 patient during the study period and due to the death of 3 patients (2 patients in the first week and one patient in the second week). *RV*_*D*_*/LV*_*D*_* ratio*, right ventricular to left ventricular diameter ratio

**Fig. 3:**
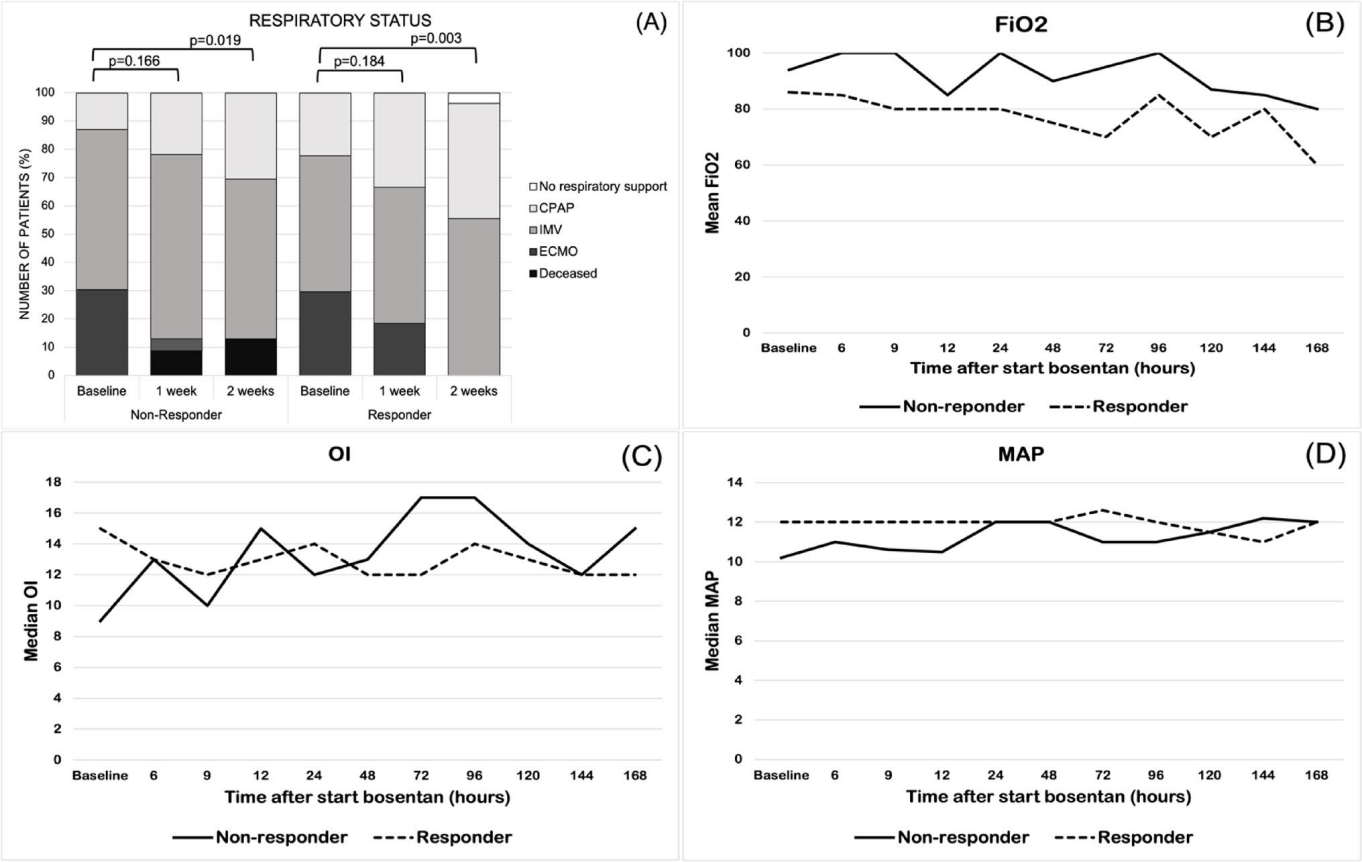
**A** The respiratory status was observed during 2 weeks after starting bosentan. Intubated patients who were also on ECMO therapy were excluded in the total count of IMV. *P*-values indicate differences in respiratory status compared to baseline. Effects of bosentan on FiO2 (**B**), oxygenation index (OI) (**C**) and mean airway pressure (MAP) (*D*) observed during 1 week after starting bosentan*.* CPAP, continuous positive airway pressure; ECMO, extracorporeal membrane oxygenation; IMV, invasive mechanical ventilation

At baseline PH was severe in 18 infants (36%), moderate in 29 infants (58%) and mild in 3 infants (6%). PH improved in 27 patients from baseline to 1 week with 8 infants (16%) showing severe, 21 infants (42%) moderate and 21 infants (42%) mild PH (*p* < 0.001). Improvement of at least one severity grade of PH was observed in 72% of patients after 2 weeks of bosentan therapy (*p* < 0.001). The distribution of PH severity between responders and non-responders was not significantly different at baseline (*p* = 0.103). Changes in PH severity and *RV*_*D*_/*LV*_*D*_ ratio over time in both groups are demonstrated in Fig. 2. Among the 29 survivors, PH severity at discharge was mild in 27 (93%) and moderate in 2 patients, while in non-survivors moderate PH was observed in 48% and severe PH in 38% of patients on their last echocardiogram. Follow-up of PH severity 12 months after discharge was available for 21 survivors. Two of the 21 patients had a remaining moderate PH 12 months after discharge, and 19 patients had no or mild PH at that time. The *RV*_*D*_/*LV*_*D*_ ratio at baseline was ≥ 1.0 in 85.7% of patients (*n* = 42, missing data in one patient). When comparing responder to non-responder, the *RV*_*D*_/*LV*_*D*_ ratio was equally distributed at baseline (*p* = 0.3). We observed a significantly better mean *RV*_*D*_/*LV*_*D*_ ratio 2 weeks after starting treatment in the responder group (responder 0.9 vs. non-responder 1.0, *p* = 0.028) (Table 2). The mean *RV*_*D*_/*LV*_*D*_ ratio after 1 week of therapy and at discharge did not differ significantly between responder and non-responder, but improved overall, with 67.4% with a *RV*_*D*_/*LV*_*D*_ ratio ≥ 1.0 and 31 of 46 patients with a *RV*_*D*_/*LV*_*D*_ ratio < 1.0. At baseline, 15 neonates (30%) received ECMO; 41 neonates (82%) were mechanically ventilated and 9 neonates (18%) received CPAP. Overall, the respiratory status improved significantly at 1 week (*p* = 0.040) and 2 weeks (*p* < 0.001) after starting bosentan. A significant improvement at 2 weeks was observed in both responders and non-responders (Fig. 3A). Of the 15 patients (30%) on ECMO therapy at baseline, 8 patients (16%) remained on ECMO after 1 week, and no patients required ECMO after 2 weeks. The proportion of newborns on mechanical ventilation decreased from 82 to 68% and 54% after 1 and 2 weeks of bosentan therapy, respectively. Seven patients were successfully extubated in the first week and six in the second week. Longitudinal changes in FiO_2_, MAP and *OI* in responders and non-responders after starting bosentan are demonstrated in Fig. 3 B, C and D. We did not observe significant changes of either parameter from baseline to 1 week. Respiratory support with FiO_2_ was higher for non-responder at baseline, but irrespective of response or non-response, the respiratory support with FiO_2_ improved in both groups over the course of 2 weeks (Fig. 3B). Overall, 38.5% of infants with an *OI* < 20 or *SOPI* < 5 (15/39) at baseline demonstrated an improved oxygenation index after commencing bosentan, while this was observed in 54.5% of infants with an *OI* ≥ 20 or *SOPI* ≥ 5 (6/9).

## Discussion

Our case series demonstrates that PH severity improved in 54% and 72% of neonates after 1 and 2 weeks of bosentan treatment, respectively. Even non-responders demonstrated a significant improvement of PH severity after 2 weeks of treatment (*p* = 0.029). The study also reveals a significant improvement in the *RV*_*D*_/*LV*_*D*_ ratio, which normalized in 55.3% after 1 week and 67.4% after 2 weeks of bosentan treatment. Based on the study design and dynamic changes in the individual treatment, it is not possible to conclusively link the improvement in the *RV*_*D*_/*LV*_*D*_ ratio to bosentan treatment alone. However, a presumed decrease in the pulmonary arterial pressure might be associated with a normalization of morphological changes such as *RV* dilatation or ventricular disproportion. This is supported by the finding that non-responders demonstrated a significantly higher *RV*_*D*_/*LV*_*D*_ ratio after 2 weeks of treatment. In terms of respiratory outcome; 8 patients showed improvement after 1 week, and an additional 9 patients improved after 2 weeks. Irrespective to responder or non-responder, the respiratory status improved in both groups over the course of 2 weeks. The need for ECMO therapy declined, with no patients receiving ECMO after 2 weeks. However, a risk of overestimating the therapeutic effect of bosentan persists, as 8 patients were still on ECMO at 1 week after starting bosentan.

To our knowledge, this is the first retrospective study investigating the effect of adjunctive bosentan therapy on the clinical course in CDH neonates. In the past, inhaled nitric oxide (iNO) and intravenous sildenafil have been widely used to treat CDH-PH, but not all patients respond sufficiently. This is often due to the underlying pathophysiology of the lung, insufficient lung recruitment, hyperinflation and differences in LV dysfunction and shunting. Current treatment of CDH-PH often relies on clinical experience rather than evidence from RCTs. Although a few case reports and exploratory RCTs on the effect of bosentan in newborns with PH have been published, these studies primarily included PPHN or preterm premature rupture of membranes (pPROM). A recent systematic review by Gao et al. [26] of nine RCTs and one retrospective study on the effect of bosentan in the treatment of PPHN revealed mixed results. While early trials showed favourable responses without any adverse events, the lack of iNO or ECMO therapy in some trials and the small sample sizes limit the strength of these findings [18]. Bosentan as an endothelin-1 receptor antagonist is however suggested as a potential therapeutic option for CDH-PH by following research showing that dysregulation of ET-A receptors and elevated ET-1 may contribute to CDH-PH. ET-1 acts as a potent pulmonary vasoconstrictor by binding to endothelin-A (ET-A) and endothelin-B (ET-B) receptors. ET-A receptors, located on vascular smooth muscle cells, cause vasoconstriction, whereas ET-B receptors mediate vasodilatation [6, 10]. De Lagausie et al. and Mous et al. have shown a significant overexpression of ET-A and ET-B receptors in the lungs of CDH patients or newborns with PPHN, with a predominance of ET-A compared to ET-B receptors [5, 11]. Additionally, higher ET-1 plasma levels have been associated with higher disease severity and poorer outcome in CDH neonates [12–14]. Contrary to the abovementioned echocardiographic findings and respiratory status, we observed only minor changes in oxygenation status and ventilator settings. Several physiological and clinical factors could explain this, such as the transition from more invasive to less invasive respiratory support, which might increase FiO_2_ requirements despite the improved PH. The *OI* as a useful marker of oxygenation status is limited to neonates receiving mechanical ventilation. When transitioning to less invasive modalities such as CPAP, this index cannot be applied. Further, the preexisting underdeveloped alveolar structures and pulmonary vasculature inherent to CDH may be limiting gas exchange capacity even if the PVR decreases. Also, prolonged mechanical ventilation can induce ventilator-associated lung injury, resulting in impaired gas exchange, even after ventilator support is de-intensified. Bosentan and other pulmonary vasodilators could potentially worsen oxygenation by reducing hypoxic pulmonary vasoconstriction (Euler-Liljestrand mechanism) [4]. These agents may increase blood flow to under-ventilated or non-aerated areas of the lung, which increases intrapulmonary shunting and reduces the efficiency of gas exchange, leading to higher FiO_2_ requirements despite improvements in PH. Thus, while PH is improved due to reduced PVR, the mismatch between ventilation and perfusion is exacerbated, contributing to the lack of improvement in oxygenation. Although the difference of patient’s characteristics including foetal markers of CDH severity were not significantly different between responders vs. non-responders, factors such as lower o/e LHR, intrathoracic liver, less isolated forms of CDH and a lower gestational age at birth might have had a cumulating effect on the disease course. This may result in a higher degree of lung hypoplasia and more aberrant pulmonary vascular development among non-responders, which could cause a lower response rate to bosentan therapy. Our dosage of 2 mg/kg/day aligns with previous literature, but recent findings suggest that up to 5 days of dosing may be required to reach therapeutic levels [18, 22, 26, 27]. This supports our observation of minimal response within the first 72 h after starting bosentan. Pharmacokinetic variability between patients may further explain the difference in response rate, as we were unable to measure bosentan levels in our cohort.

The observation that significantly fewer responders were discharged on bosentan compared to non-responders can likely be explained by the practice of weaning responders to sildenafil monotherapy after achieving clinical improvement. In the responder group, bosentan was discontinued earlier following significant improvements in clinical condition and echocardiographic findings. In contrast, bosentan was often continued in non-responders due to a lack of improvement or was administered until death. A notable limitation of this study is that bosentan therapy was not consistently discontinued in cases where no response was observed after 2 weeks of treatment. The duration of ECMO support, mechanical ventilation, and hospital stay did not differ significantly between responders and non-responder. This lack of significant difference could be influenced by other contributing factors beyond elevated PVR, such as lung injury from prolonged ventilation and lung hypoplasia, opioid withdrawal syndrome or feeding difficulties.

This cohort was highly selective, as only 54 of 210 (25.7%) CDH newborns at our institution received bosentan, resulting in a relatively high mortality rate of 42% in the study cohort, despite a reduction in ECMO use. This discrepancy indicates that mortality was primarily caused by underlying pulmonary pathophysiology or other causes of death, for which extended ECMO support would not have improved survival. Notably, no patients in this cohort required a second ECMO run. The elevated mortality rate among the 27 patients (54%) who responded favourably to bosentan therapy may be attributed to factors unrelated to PH, including chronic ventilator-induced lung injury, lung hypoplasia or cardiac dysfunction. Importantly, responders and non-responders were classified based on improvement of PH after 1 week of therapy; later improvements, which may take longer to manifest, were not evaluated. In an attempt to quantify *LV* dysfunction, we compared the *LV-GLS* between responders and non-responders at baseline and after 1 week of treatment. Although an abnormal *LV-GLS* demonstrated *LV* dysfunction in 25.9% of responders and 39.1% of non-responders at baseline, this difference was not statistically significant. Whether, *LV* dysfunction may contribute to non-responding to bosentan in CDH neonates, as it has been described in other pulmonary vasodilators such as sildenafil or iNO, needs to be investigated in future trials. However, in most patients *LV* dysfunction was not severe, presumably not contributing to increased mortality. Finally, 44% of the neonates in this cohort had non-isolated CDH with varying comorbidities, such as DiGeorge syndrome, ventricular septal defect or pulmonary sequestration, all of which could have contributed to the elevated mortality rate.

Common adverse effects of bosentan include elevated LFT, liver failure, anaemia, leukopenia and thrombocytopenia [28]. However, it remains unclear if this was drug-related or due to other factors, such as sepsis or liver bleeding. No patients discontinued treatment due to adverse effects, suggesting a consistent safety profile with previous studies [18–22]. This study is limited by its retrospective design, small sample size and single-centre setting, which may reduce statistical significance of our findings. Additionally, we did not routinely measure ET-1 level or cardiac biomarkers such as proBNP, which have been associated with disease severity in CDH neonates [14, 29]. Furthermore, the lack of a control group receiving alternative therapies or no bosentan therapy limits our ability to compare outcomes directly. Neonates in this study received bosentan predominantly for moderate to severe PH, which introduces potential selection bias. Differences in disease severity or treatment eras likely influenced the decision to use bosentan, making direct comparisons with non-treated patients difficult and subject to confounding factors. Another important limitation is the potential for natural improvement in PH and respiratory status in some patients, independent of bosentan therapy. This possibility highlights the inherent limitations of retrospective case series in determining treatment efficacy. Although bosentan is generally well-absorbed, variability in feeding schedules and gastrointestinal function in neonates with CDH may have impacted the bioavailability of the drug, leading to inconsistent responses among the study population. Lastly, the timing of bosentan initiation could have influenced response rates, particularly in relation to the surgical CDH repair. While this cohort received bosentan early, the exact timing relative to hernia repair (pre- or post-repair) was not standardized and may have contributed to differences in outcomes. Earlier initiation of bosentan, potentially before repair, may have the potential to reduce the need for ECMO more, though further investigation is needed to clarify this hypothesis.

## Conclusion

Based on our findings, the use of enteral adjunctive bosentan was associated with reduced PH in 54% of newborns with CDH-PH after 1 week and in 72% after 2 weeks of treatment. Additionally, 34% of patients showed improvements in respiratory status within 2 weeks. However, oxygenation status remained largely unchanged over the 2-week period, possibly influenced by changes in respiratory support. The variations in response rates for PH, respiratory status and oxygenation warrant further investigation. More large-scale RCTs are needed to elucidate the potential benefits of bosentan in the treatment of CDH-PH.

## Data Availability

No datasets were generated or analysed during the current study.
